# Subhepatic appendicitis mimicking biliary disease: diagnostic pitfalls and laparoscopic management

**DOI:** 10.1093/jscr/rjag270

**Published:** 2026-04-20

**Authors:** Cielo S Silva-Ramos, Kevin J Fuentes-Calvo, Jorge Leal-Hidalgo, José Arturo Vergara-Aceves, Diego García-Vivanco, Alejandro Díaz Girón-Gidi, Eduardo Villegas-Tovar

**Affiliations:** Department of General Surgery, Hospital Médica Sur, Puente de Piedra 150, 140550 Mexico City, Mexico; Department of General Surgery, Hospital Médica Sur, Puente de Piedra 150, 140550 Mexico City, Mexico; Department of General Surgery, Hospital Médica Sur, Puente de Piedra 150, 140550 Mexico City, Mexico; Department of General Surgery, Hospital Médica Sur, Puente de Piedra 150, 140550 Mexico City, Mexico; Department of General Surgery, Hospital Médica Sur, Puente de Piedra 150, 140550 Mexico City, Mexico; Department of Gastrointestinal Minimally Invasive Surgery, Hospital Médica Sur, Puente de Piedra 150, 140550 Mexico City, Mexico; Department of General Surgery, Hospital Médica Sur, Puente de Piedra 150, 140550 Mexico City, Mexico; Department of Gastrointestinal Minimally Invasive Surgery, Hospital Médica Sur, Puente de Piedra 150, 140550 Mexico City, Mexico; Department of General Surgery, Hospital Médica Sur, Puente de Piedra 150, 140550 Mexico City, Mexico; Department of Gastrointestinal Minimally Invasive Surgery, Hospital Médica Sur, Puente de Piedra 150, 140550 Mexico City, Mexico

**Keywords:** subhepatic appendicitis, minimally invasive surgery, anatomical variation, acute appendicitis, biliary disease mimicker

## Abstract

The subhepatic appendix is a rare anatomical variant, accounting for up to 0.06% of cases of acute appendicitis. Its atypical presentation, low incidence, and nonspecific clinical features pose a significant diagnostic and therapeutic challenge for surgeons. Laparoscopic appendectomy remains the treatment of choice; however, an open approach may be warranted in cases of severe sepsis, perforation, or extensive intra-abdominal adhesions. We present a case series describing the diagnostic and surgical management of subhepatic appendicitis in a tertiary referral center in Mexico City.

## Introduction

Acute appendicitis remains one of the most common surgical emergencies worldwide, with a reported lifetime incidence of ~7%–8%. In contrast, the subhepatic appendix is an uncommon anatomical variant, with an estimated annual incidence of 0.06 per 100 000 inhabitants. This condition is primarily attributed to congenital adhesions resulting from abnormal embryological development of the cecum and appendix [1, 2]. Owing to its atypical and nonspecific clinical presentation, the diagnosis of subhepatic appendicitis represents a significant clinical challenge. Currently, laparoscopic surgery is the preferred therapeutic approach, as it offers several well-established advantages; however, its main limitations include the availability of specialized equipment and, more importantly, the surgeon’s expertise. Diagnostic delays related to this unusual presentation may result in disease progression, leading to complications such as perforation, intra-abdominal abscess formation, generalized peritonitis, and even life-threatening scenarios [3].

## Case report

### Case 1

**Figure 1:**
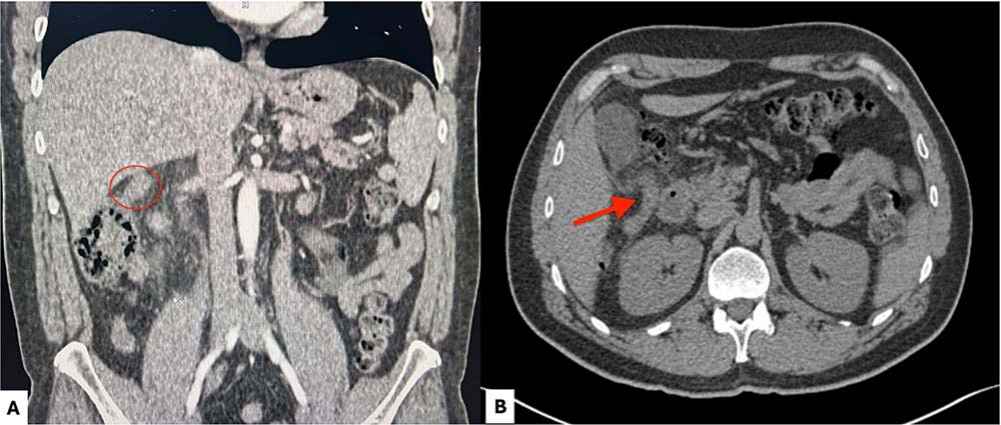
Contrast-enhanced abdominal computed tomography. (A) Coronal view showing a subhepatic appendix (red circle) measuring 12 mm in diameter. (B) Axial view demonstrating a subhepatic appendix (red arrow) with mural thickening, increased enhancement, and surrounding periappendiceal fat stranding.

**Figure 2:**
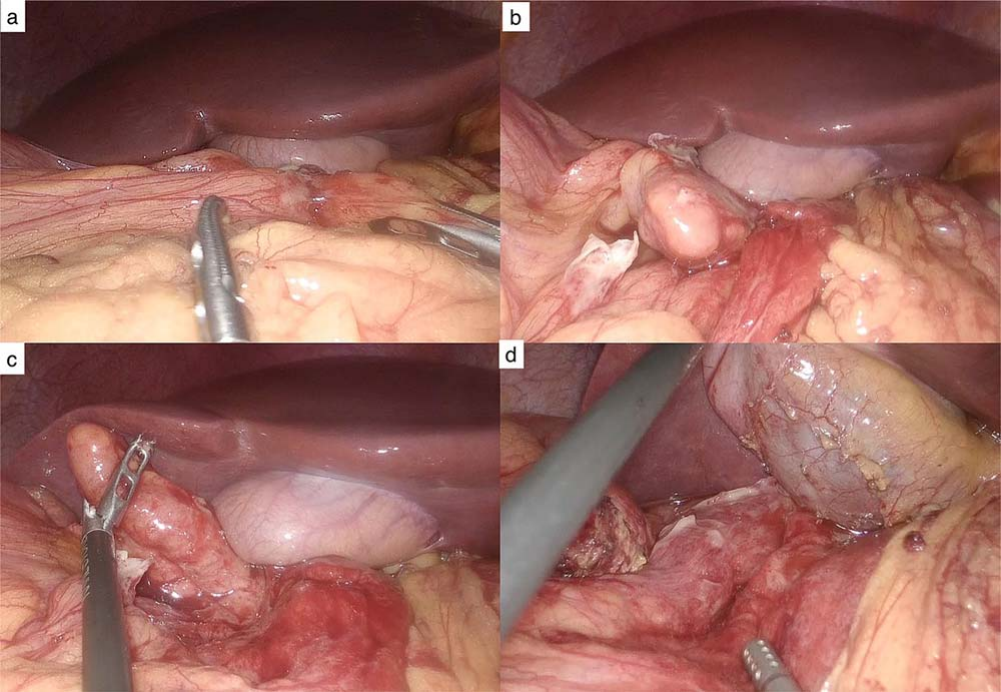
Laparoscopic findings**.** (a) Inflammatory plastron involving the gallbladder. (b) After dissection, the tip of the appendix is identified anterior to the gallbladder. (c) Subhepatic appendix located anterior to the gallbladder. (d) Appendiceal bed following appendectomy.

A 45-year-old male presented with respiratory symptoms and colicky epigastric abdominal pain radiating to the right upper quadrant, exacerbated by cholecystokinetic foods, and associated with nausea, vomiting, and chills. Physical examination revealed right upper quadrant tenderness, a positive Murphy’s sign, and rebound tenderness. Laboratory tests demonstrated leukocytosis with neutrophilia, elevated C-reactive protein levels, and a cholestatic pattern. Abdominal ultrasonography of the liver and biliary tract showed no abnormalities. The patient tested positive for SARS-CoV-2 infection. Due to persistent and worsening right upper quadrant pain, abdominal computed tomography was performed, revealing acute appendicitis (Fig. 1). Surgical management was undertaken via laparoscopy, with trocar placement at the transumbilical, left flank (midclavicular line), and suprapubic sites. Intraoperative findings included a subhepatic appendix densely adherent to the gallbladder and duodenum, with abundant fibrinopurulent exudate (Fig. 2a–d). The postoperative course was uneventful, and the patient was discharged without complications.

### Case 2

A 46-year-old male presented with watery diarrhea without mucus or blood, accompanied by colicky right upper quadrant abdominal pain, nausea without vomiting, fever (38°C), chills, conjunctival icterus, and dark urine. On physical examination, the abdomen was tender to palpation in the right upper quadrant and right flank, with positive Murphy’s sign and rebound tenderness. Laboratory studies revealed leukocytosis with neutrophilia and a cholestatic biochemical profile. Abdominal ultrasonography demonstrated an inflammatory mass in the subhepatic region, and contrast-enhanced abdominal computed tomography confirmed acute appendicitis. Based on these findings, laparoscopic appendectomy with partial cecectomy was performed. Four trocars were placed (transumbilical, subxiphoid, right upper quadrant midclavicular line, and right flank anterior axillary line). Intraoperatively, an inflammatory phlegmon adherent to the hepatic bed was identified, along with an 8 × 2 cm appendix with proximal third perforation and a preserved appendiceal base. The patient had a favourable postoperative recovery and was discharged without further complications.

### Case 3

A 40-year-old male presented with left upper quadrant abdominal pain radiating in a belt-like pattern to the ipsilateral lumbar region, worsened by positional changes, and associated with nausea and vomiting. Physical examination revealed abdominal distension, tympanism, and tenderness in the right upper quadrant and right flank, with positive Murphy’s sign and rebound tenderness. Laboratory evaluation showed leukocytosis with neutrophilia, elevated acute-phase reactants, and increased alkaline phosphatase levels. Abdominal ultrasonography suggested acute cholecystitis. Surgical management was performed laparoscopically, revealing a subhepatic appendix in close contact with the gallbladder, measuring 11 × 1.5 × 1 cm, with an edematous and hyperemic wall covered by fibrinopurulent exudate. The gallbladder was edematous (11 × 5 × 4 cm) and contained multiple gallstones. Four trocars were placed in the standard configuration for laparoscopic cholecystectomy (transumbilical, subxiphoid, right upper quadrant midclavicular line, and right flank anterior axillary line). The postoperative course was favorable, and the patient was discharged without complications.

## Discussion

Acute appendicitis remains one of the most common surgical emergencies worldwide and represents a significant burden on modern healthcare systems. Regarding unusual appendiceal presentations, the first case of subhepatic appendicitis was reported by King in 1955; however, the earliest description of a subhepatic appendix dates back to 1863 in an autopsy report. The appendix is a vestigial organ located on the posteromedial aspect of the cecum, ~2.5 cm below the ileocecal valve, and is the only organ in the human body without a fixed anatomical position. The subhepatic location is determined by abnormal embryological migration of the cecum: during late embryogenesis, the cecum initially ascends from the umbilical region toward the stomach and subsequently rotates counterclockwise from left to right, reaching a subhepatic position beneath the right hepatic lobe, where this stage is completed around the fifth month of gestation. Normally, the descent of the cecum begins after the fifth month and its definitive position in the right iliac fossa is completed by the eighth month of gestation [4].

In a series of 7210 patients with appendicitis between 1992 and 2006, six cases of subhepatic appendix were identified, corresponding to an incidence of 0.06%. Patients typically presented with fever, nausea, and right upper quadrant tenderness, often mimicking acute cholecystitis [5].

From a diagnostic perspective, abdominal ultrasonography is usually the first imaging modality employed; however, it has limited sensitivity and is highly operator-dependent. In contrast, computed tomography is considered the most reliable modality for identifying subhepatic appendicitis, demonstrating high sensitivity (100%), specificity (95%), and diagnostic accuracy (98%) for acute appendicitis [6–8]. Delayed diagnosis of subhepatic appendicitis may lead to disease progression and severe complications, including appendiceal perforation and intra-abdominal abscess formation.

For definitive management of these uncommon presentations, laparoscopic appendectomy is considered a safe and effective treatment for non-perforated subhepatic appendicitis [6, 9]. Numerous studies have demonstrated the superiority of laparoscopic over open appendectomy, including a lower incidence of postoperative ileus, reduced risk of surgical site infection, shorter hospital stay, decreased postoperative analgesic requirements, reduced adhesion formation, and the advantages of a minimally invasive approach. Nevertheless, surgeon experience remains a critical determinant of successful laparoscopic outcomes [1, 10].

Some authors recommend a three-port technique for appendectomy in atypical appendiceal locations, consisting of a 10-mm supraumbilical port, a 10-mm epigastric port, and a 5-mm right flank port [11]. Alternatively, an open approach may be considered, with a midline incision recommended for its versatility and superior exposure, particularly in patients with severe septic conditions, perforation, or extensive adhesions [6].

## Conclusion

The subhepatic appendix is a rare condition resulting from abnormal cecal malrotation during embryogenesis. Surgeons should consider this entity in the differential diagnosis of right upper quadrant pain and possess the diagnostic and technical skills required for its laparoscopic management, which has been shown to be the treatment of choice.

Early diagnosis and an appropriate clinical approach, including consideration of atypical differential diagnoses, are essential for identifying subhepatic appendicitis and achieving successful laparoscopic treatment. Prompt surgical intervention facilitates rapid patient recovery and shortens hospital stay.
